# New tale on LianHuaQingWen: IL6R/IL6/IL6ST complex is a potential target for COVID-19 treatment

**DOI:** 10.18632/aging.203666

**Published:** 2021-11-03

**Authors:** Zhao Tianyu, Cui Xiaoli, Wang Yaru, Zhang Min, Yue Fengli, He Kan, Chen Li, Li Jing

**Affiliations:** 1 Department of Pharmacology, College of Basic Medical Sciences, Jilin University, Changchun, Jilin Province 130021, People’s Republic of China

**Keywords:** LianHuaQingWen (LHQW), COVID-19, IL6R/IL6/IL6ST complex, quercetin, AGE-RAGE signaling pathway

## Abstract

LianHuaQingWen (LHQW) improves clinical symptoms and alleviates the severity of COVID-19, but the mechanism is unclear. This study aimed to investigate the potential molecular targets and mechanisms of LHQW in treating COVID-19 using a network pharmacology-based approach and molecular docking analysis. The main active ingredients, therapeutic targets of LHQW, and the pathogenic targets of COVID-19 were screened using the TCMSP, UniProt, STRING, and GeneCards databases. According to the “Drug-Ingredients-Targets-Disease” network, Interleukin 6 (IL6) was identified as the core target, and quercetin, luteolin, and wogonin as the active ingredients of LHQW associated with IL6. The response to lipopolysaccharide was the most significant biological process identified by gene ontology enrichment analysis, and AGE-RAGE signaling pathway activation was prominent based on the interaction between LHQW and COVID-19. Protein-protein docking analysis showed that IL6 receptor (IL6R)/IL6/IL6 receptor subunit beta (IL6ST) and Spike protein were mainly bound via conventional hydrogen bonds. Furthermore, protein-small molecule docking showed that all three active ingredients could bind stably in the binding model of IL6R/IL6 and IL6ST. Our findings suggest that LHQW may inhibit the lipopolysaccharide-mediated inflammatory response and regulate the AGE-RAGE signaling pathway through IL6. In addition, the N-terminal domain of the S protein of COVID-19 has a good binding activity to IL6ST, and quercetin and wogonin in LHQW may affect IL6ST-mediated IL6 signal transduction and a large number of signaling pathways downstream to other cytokines by directly affecting protein-protein interaction. These findings suggest the potential molecular mechanism by which LHQW inhibits COVID-19 through the regulation of IL6R/IL6/IL6ST.

## INTRODUCTION

COVID-19 has spread across the globe since its outbreak at the end of 2019 due to its high susceptibility and transmissibility [1]. Patients often display symptoms of fever, dry cough, fatigue, and even lung inflammation and infiltration, as well as systemic cytokine storms [2]. All countries worldwide are trying to develop drugs to treat this disease. In the absence of a specific cure for COVID-19, traditional Chinese medicine (TCM) plays an important role in inhibiting inflammation, preventing the development of disease, and shortening the treatment cycle because it has multiple components and targets, broad-spectrum antibacterial and antiviral activity, and can improve immunity.

LianHuaQingWen (LHQW), a type of TCM used to treat respiratory diseases that are identified during public health events, was developed based on the experience of well-known Chinese doctors in Han, Ming, and Qing dynasties for treating cold, influenza, and other exogenous febrile diseases combined with modern pharmacological research. According to the Pharmacopeia of the People’s Republic of China 2015 Edition [3], LHQW is a compounded medication (Supplementary Table 1) composed of 13 components, including primary, secondary, and adjuvant components, and the toxicity of TCM is generally controllable. It can clear away the “lung heat,” inhibit bacterial infection, enhance immunity, reduce inflammation and fever, relieve cough, reduce sputum, reduce high fever, and alleviate aversion to cold, muscle pain, headache, sore throat, and other symptoms of heat toxicity.

LHQW has a history of over 2,000 years of being used as a TCM medication during epidemic diseases. Previous studies have shown that LHQW had a broad-spectrum of antiviral effects targeting many viruses, including SARS coronavirus (SARS-CoV), Middle East respiratory syndrome coronavirus (MERS-CoV), H1N1, H3N2, and H7N9. It also exhibits antibacterial, anti-inflammatory, antipyretic, cough-relieving, expectorant, immunity-regulating, and other systematically intervening properties [4–5]. Since the COVID-19 outbreak, LHQW has been listed as the recommended drug during the observation period of COVID-19 in more than 20 national and local COVID-19 diagnostic and treatment plans [6]. Owing to the participation of the Chinese medical team in local COVID-19 treatments in Italy and other countries, LHQW has obtained registration approval in six countries, including Brazil, Romania, Thailand, Ecuador, Singapore, and China, as well as Hong Kong and Macao SAR. On April 14, 2020, during the Press Conference of the Joint Prevention and Control Mechanism of the State Council, it was announced that LHQW could significantly relieve cough, fever, and fatigue caused by COVID-19 and could effectively reduce the rate of mild-to-severe symptoms; thus, it could be used for the routine treatment of COVID-19. In addition, with the approval of the National Medical Products Administration (NMPA), “In the routine treatment of COVID-19, it can be used for fever, cough, and fatigue caused by the mild and common type” was added in “Functions and indications” in the drug instructions, and “The course of treatment for mild and common COVID-19 is 7–10 d” was added in “Usage and dosage” [7]. A recent study by Hu et al. [2] showed that the recovery rate for COVID-19 patients treated with LHQW was significantly higher than that in the control group because it improved the clinical symptoms and reduced the severity of the disease. It is suggested that the drug has clinical significance for the treatment of COVID-19, but the mechanism of LHQW in treating COVID-19 has not yet been identified.

TCM prescriptions are known for being multi-component and multi-target, and involved in systemic regulation. Therefore, network pharmacology is an effective method for studying the mechanisms of action of these prescriptions. Yan et al. [8] and Wang et al. [9] have used network pharmacology and molecular docking technology to show that LHQW capsule could provide improved efficacy as a cure for COVID-19 through its multi-herb, multi-target, multi-signaling pathway and multi-biological function. The Spike (S) protein, including the S1 and S2 subunits, of COVID-19 is the key protein that mediates the entry of the virus into the host cell. The S protein promotes the fusion of the viral envelope with the target cell membrane or endosome membrane that allows the viral nucleic acid to enter the cell [10–11]. However, it is unclear whether LHQW can interfere with this process. This study aimed to investigate the mechanism of LHQW protective action during COVID-19 infection using network pharmacology-based approach and molecular docking analysis.

## RESULTS

### Results of network pharmacology analysis of LHQW protective action during COVID-19 infection

#### LHQW ingredients-targets network and protein-protein interaction network including COVID-19 pathogenic targets, LHQW therapeutic targets, junction targets of COVID-19, and LHQW

GeneCards, TCMSP, and UniProt databases were used to screen 277 COVID-19 pathogenic targets. In addition, a network of 80 LHQW active ingredients and 234 therapeutic targets was constructed after screening (Supplementary Figure 1A and Supplementary Table 2).

To analyze the association between targets, 277 COVID-19 pathogenic targets and 234 therapeutic targets were imported into the STRING database respectively. Protein-protein interaction (PPI) networks of 266 COVID-19 pathogenic targets and 233 LHQW therapeutic targets were obtained after removing duplicate values separately. Cytoscape was used to optimize the two networks and compute the values of degrees of network connections. Based on the maximum degree value, TNF and AKT1 were respectively identified as the core targets in the network of COVID-19 pathogenic targets (Supplementary Figure 2) and the network of LHQW therapeutic targets (Supplementary Figure 1B). In addition, secondary and tertiary targets were identified based on the values of their degree (in decreasing order). The darkness of the color and size of the circle are positively correlated with the role of the target in the network.

**Figure 1:**
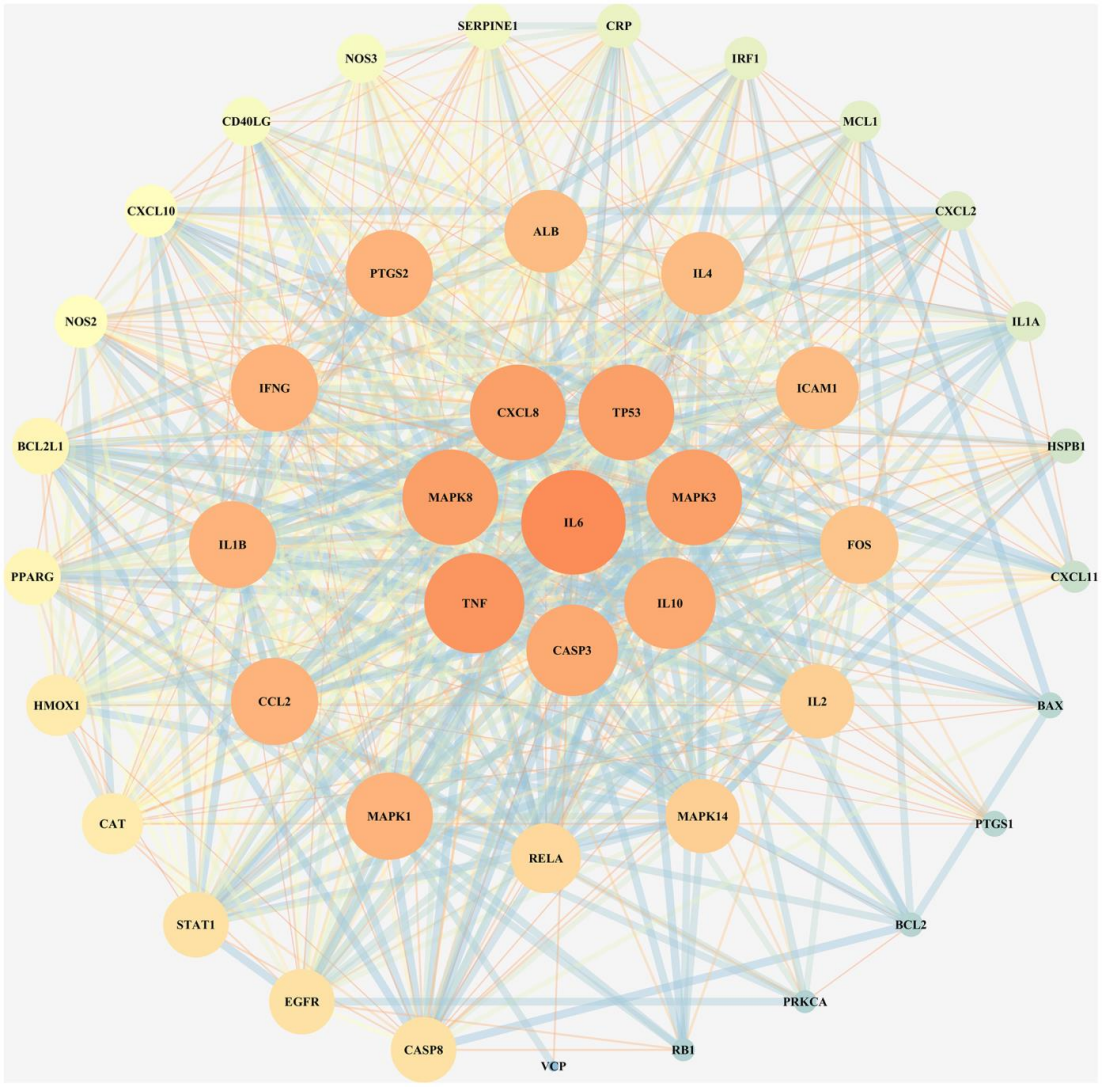
PPI network for junction targets of COVID-19 and LHQW.

To investigate the reflective relationship between COVID-19 and LHQW, the potential targets of COVID-19 were mapped with those of LHQW to obtain 45 junction targets. Using these targets, the PPI network of LHQW in treating COVID-19 was constructed using STRING and Cytoscape. Interleukin 6 (IL6) was detected as the core target according to a degree ≥41, and seven secondary targets, including TNF, MAPK8, CXCL8, TP53, MAPK3, IL10, and CASP3, were detected based on 38 ≤ degree <41. In terms of 33 ≤ degree < 38, 12 tertiary targets were further identified, including MAPK1, CCL2, IL1B, IFNG, PTGS2, ALB, IL4, ICAM1, FOS, IL2, MAPK14, and RELA. (See Figure 1).

#### Gene-Concept network of gene ontology enrichment analysis and Kyoto Encyclopedia of Genes and Genomes pathway analysis of junction targets of COVID-19 and LHQW

**Figure 2:**
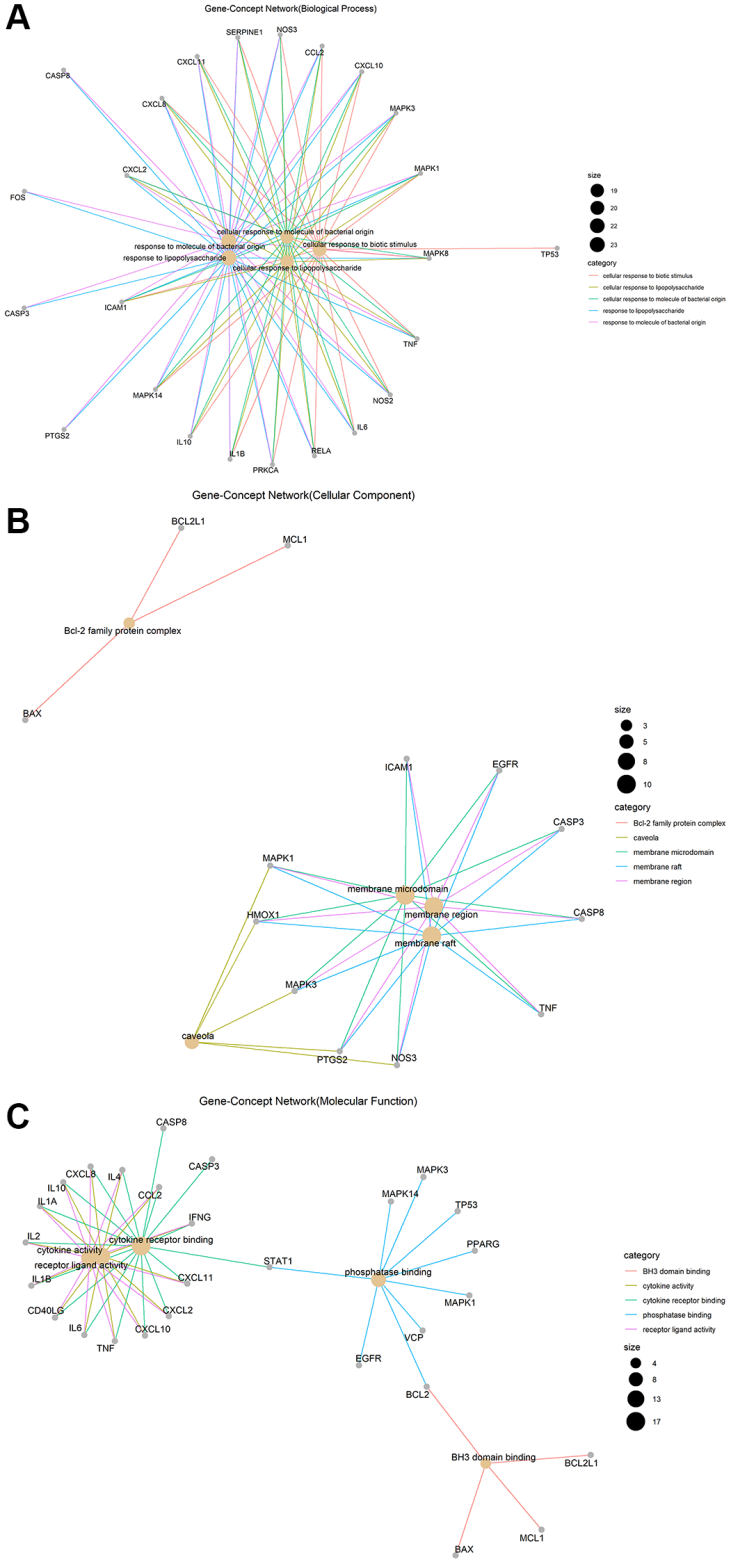
**Gene-Concept network of GO enrichment analysis of junction targets of COVID-19 and LHQW.** (**A**) Biological processes. (**B**) Cellular components. (**C**) Molecular functions.

**Figure 3:**
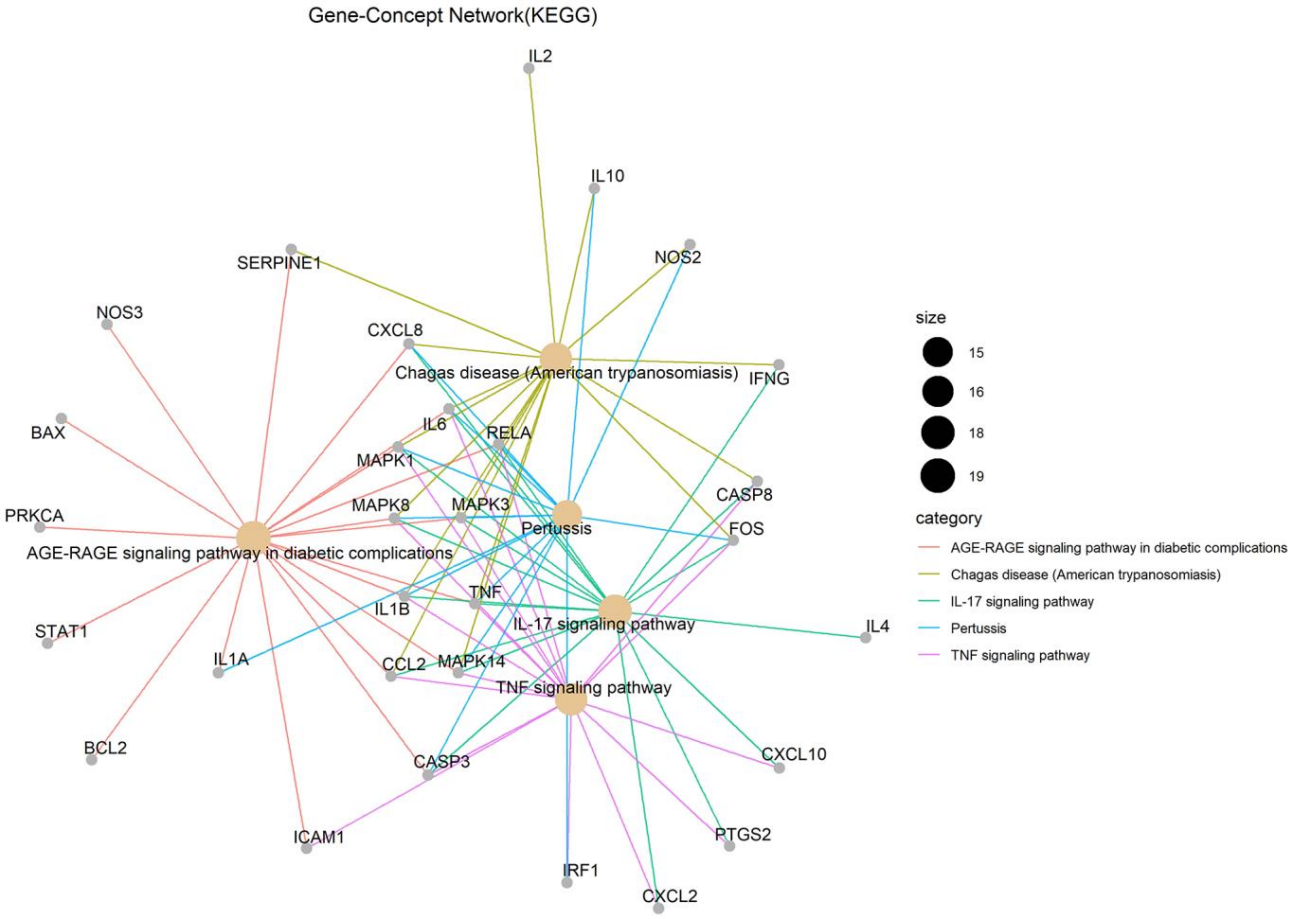
KEGG pathways.

Gene ontology (GO) enrichment analysis and Kyoto Encyclopedia of Genes and Genomes (KEGG) pathway analysis identified 2,274 biological processes (*P* ≤ 0.05), 50 cellular components (*P* ≤ 0.05), 137 molecular functions (*P* ≤ 0.05), and 152 KEGG pathways (*P* ≤ 0.05) in which junction targets of COVID-19 and LHQW were involved, separately. In terms of *P* value (in increasing order), the five most significant biological processes (Figure 2A), cellular components (Figure 2B), molecular functions (Figure 2C), and KEGG pathways (Figure 3) were screened to build the Gene-Concept network respectively.

#### Results of core target IL6 for LHQW in treating COVID-19

##### IL6 was involved in the most significant GO enrichment and KEGG pathways

**Figure 4:**
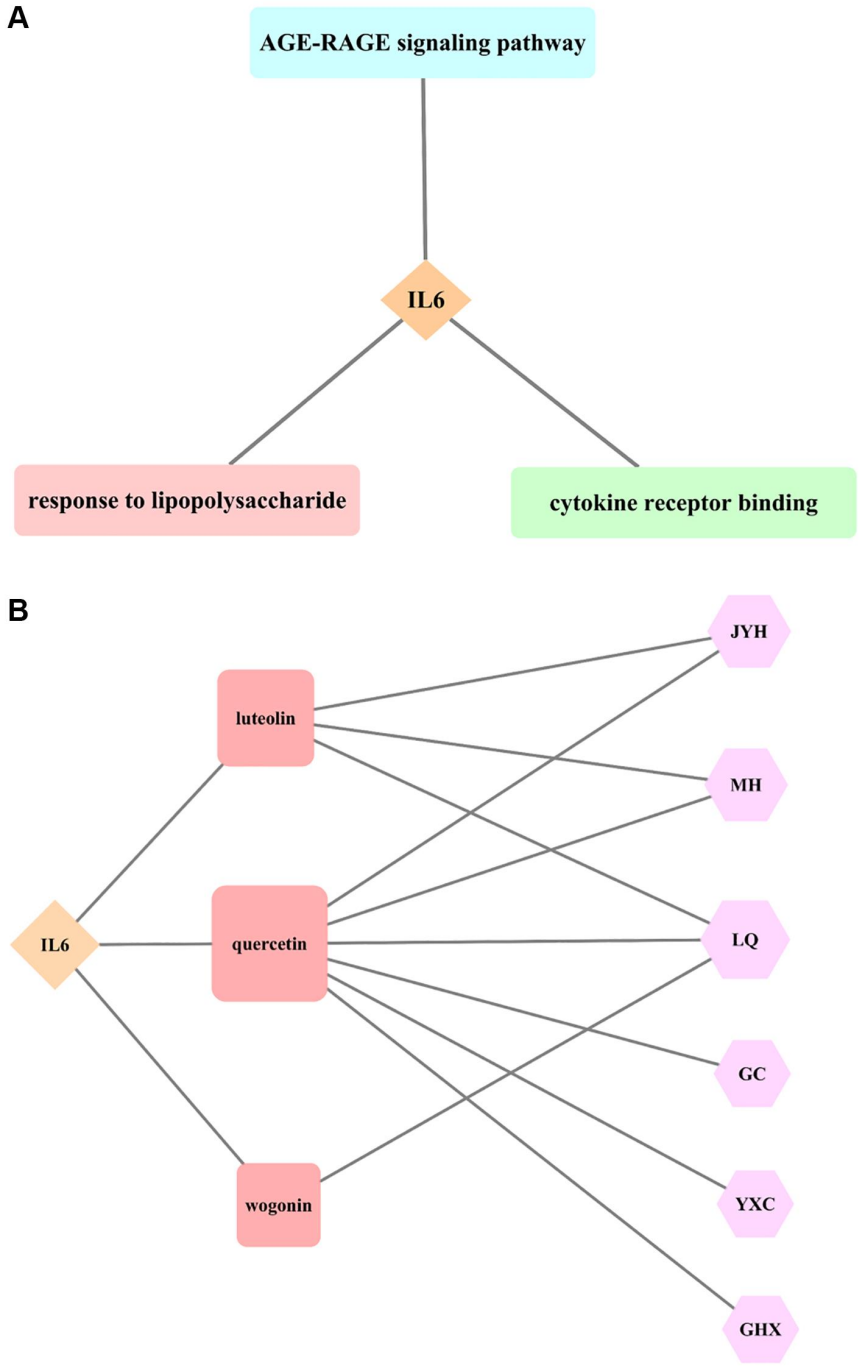
**Results of core target IL6 for LHQW in treating COVID-19.** (**A**) The most significant GO enrichment and KEGG pathway analyses. (**B**) Effective active ingredients.

The analysis of IL6 as the core target of LHQW in treating COVID-19 showed that there were five biological processes that included IL6 in the top five biological processes in which the junction targets were involved (*P* ≤ 0.05). Response to lipopolysaccharide (LPS) was identified as the biological process in which IL6 was most actively involved (*P* ≤ 2.23 × 10^−25^). There were no cellular components with IL6 involvement among the first five cellular components in which the junction targets of COVID-19 and LHQW were included. There were three molecular functions with IL6 involvement among the first five molecular functions in which the junction targets of COVID-19 and LHQW were involved (*P* ≤ 0.05). According to *P* ≤ 4.15 × 10^−17^, the most significant molecular function with IL6 involvement was cytokine receptor binding. There were 5 KEGG pathways with IL6 involvement in the first 5 KEGG pathways in which the junction targets of COVID-19 and LHQW were involved (*P* ≤ 0.05). IL6 involvement was most active in the AGE-RAGE signaling pathway (*P* ≤ 2.36 × 10^−23^) (Figure 4A).

##### AGE-RAGE signaling pathway in diabetic complications

The AGE-RAGE signaling pathway in diabetic complications contained 19 targets. In addition to the core target IL6 (red), there were five secondary targets (pink), including TNF, CXCL8, MAPK8, MAPK3, and CASP3, and six tertiary targets (seashell), including IL1B, CCL2, MAPK1, ICAM1, MAPK14, and RELA, as well as seven quaternary targets (seashell), including STAT1, NOS3, SERPINE1, IL1A, BAX, BCL2, and PRKCA, indicating that the effect of LHQW on the prevention and control of COVID-19 was closely related to the regulation of various targets (Supplementary Figure 3).

##### Effective active ingredients of IL6

The results of this study suggest that the effective active ingredients, including luteolin (Lonicerae Japonicae Flos, Ephedra Herba, Forsythiae Fructus), quercetin (Lonicerae Japonicae Flos, Ephedra Herba, Forsythiae Fructus, licorice, Houttuyniae Herba, Pogostemon Cablin (Blanco) Benth.), and wogonin (Forsythiae Fructus) are all connected with the core target IL6, indicating that the effect of LHQW on the prevention and treatment of COVID-19 is closely related to these three active ingredients (Figure 4B).

### Verification of molecular docking for LHQW in treating COVID-19 by the results of network pharmacology

#### Protein-protein docking analysis of IL6R/IL6/IL6ST and COVID-19-Spike interactions

Protein-protein docking between COVID-19-Spike and IL6 receptor (IL6R)/IL6/IL6 receptor subunit beta (IL6ST) was simulated to verify whether IL6, the core target identified through our network pharmacology-based approach, was a pathogenic target. The results showed that IL6R/IL6/IL6ST (PDB ID: 1P9M) had high level of CDOCKER interaction energy with the Spike protein of COVID-19 (PDB ID: 6VXX). The maximum ZDOCK score was 1955.699.

##### The binding model of IL6R/IL6/IL6ST and Spike

**Figure 5:**
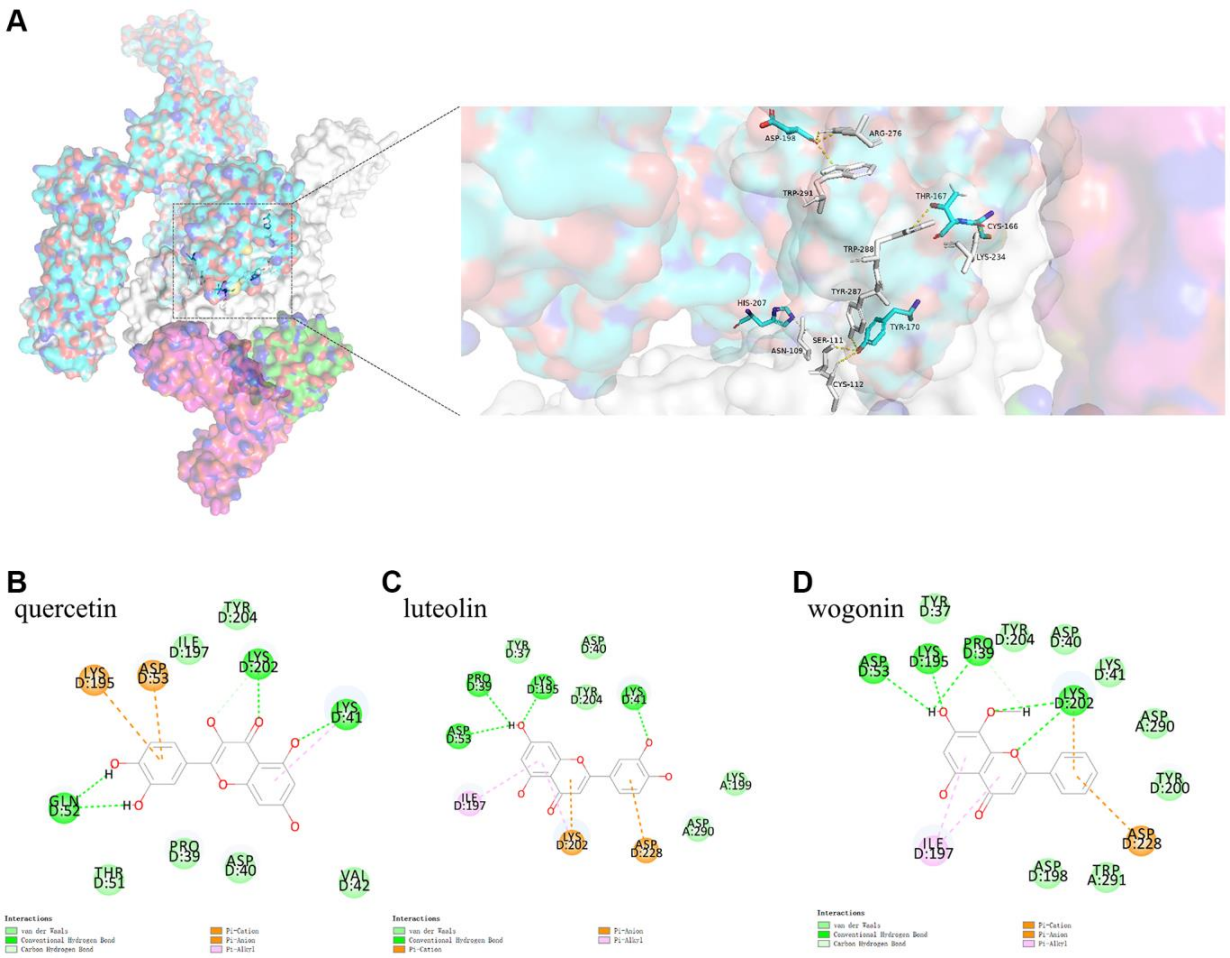
(**A**) shows the binding model of IL6ST and Spike. Spike protein (blue), IL6ST (white), IL6 (green), IL6R (pink) can be seen from the Figure. (**B**–**D**) are the docking models of quercetin, luteolin, wogonin with the interaction center of IL6ST and Spike of 1P9M-6VXX complex, respectively.

To simulate the process by which COVID-19 attaches to the host cell receptor, Pymol was used to analyze the binding model of IL6R/IL6/IL6ST and Spike. This analysis revealed that conventional hydrogen bonds occurred between the IL6R/IL6/IL6ST amino acid residues Arg276 (IL6ST), Trp291 (IL6ST), Trp288(IL6ST), Lys234 (IL6ST), Trp287 (IL6ST), Ser111 (IL6ST), Cys112 (IL6ST), Asn109 (IL6ST), and the Spike amino acid residues Asp198, Asp198, Thr167, Cys166, Tyr170, Tyr170, Tyr170, and His207, respectively (Figure 5A).

##### The binding model between IL6R/IL6 and IL6ST

**Figure 6:**
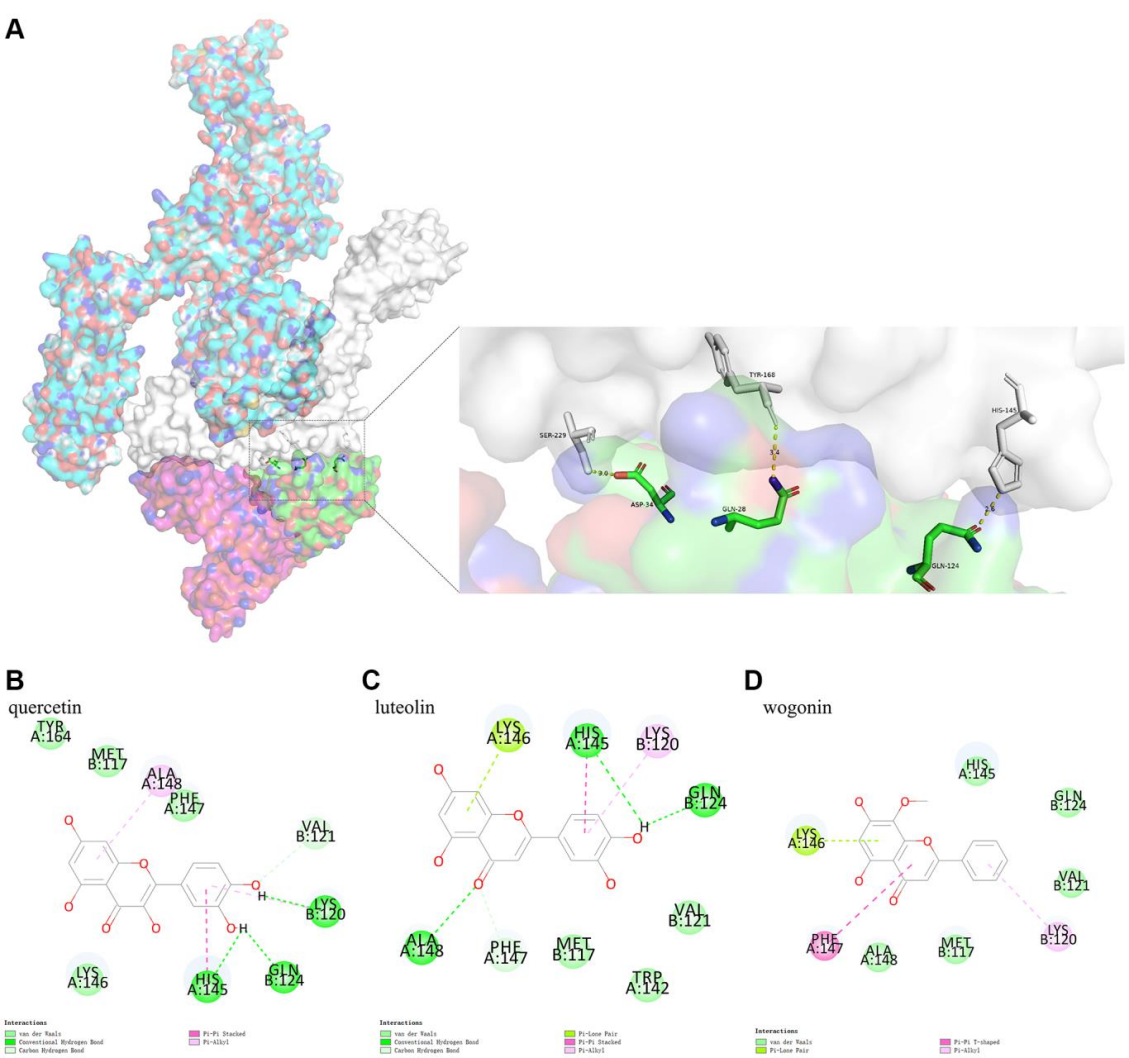
(**A**) shows the binding model of IL6R/IL6 and IL6ST. Spike protein (blue), IL6ST (white), IL6 (green), IL6R (pink) can be seen from the Figure. (**B**–**D**) are the docking models of quercetin, luteolin, wogonin with the interaction center of IL6R/IL6 and IL6ST of 1P9M-6VXX complex, respectively.

Due to its biological characteristics, IL6R binds to IL6 and then binds to IL6ST with high affinity, thus inducing signal transduction process. This study analyzed the binding model of IL6R/IL6 and IL6ST using Pymol. The results indicated that IL6ST amino acid residues Ser229, Tyr168, and His145 formed conventional hydrogen bonds with IL6R/IL6 amino acid residues Asp34 (IL6), Gln28 (IL6), and Gln124 (IL6), respectively (Figure 6A).

##### Protein-small molecule docking analysis

The activity of the three active ingredients obtained through our network pharmacology-based approach was validated through protein-small molecule docking analysis.

##### Protein-small molecule docking analysis based on the binding model of IL6R/IL6 and IL6ST

Protein-small molecule docking, based on the binding model of IL6R/IL6 and IL6ST, was performed to determine if the three active ingredients affected binding between IL6R/IL6 and IL6ST. We found that the CDOCKER interaction energy values of quercetin, luteolin, and wogonin docking with the interaction center of IL6R/IL6 and IL6ST (1P9M-6VXX complex) were −26.1801 kcal mol^−1^, −24.3014 kcal mol^−1^, and −24.9362 kcal mol^−1^, respectively. The binding model analyzed by Pymol showed that there was a conventional hydrogen bond between quercetin and Gln124 (IL6R/IL6) of the 1P9M-6VXX complex, and conventional hydrogen bond and Pi-Pi stacking could be found between quercetin and His145 (IL6ST) of the 1P9M-6VXX complex. It should also be noted that Gln124 (IL6R/IL6) and His145 (IL6ST) are the key amino acids required for stable binding between IL6R/IL6 and IL6ST (Figure 6B–6D).

##### Protein-small molecule docking analysis based on the binding model of IL6R/IL6/IL6ST and Spike

To investigate whether the three active ingredients affected COVID-19 attachment to the host cell receptors, protein-small molecule docking analysis was used based on the binding model of IL6R/IL6/IL6ST and Spike. This analysis showed that the CDOCKER interaction energy levels of the 1P9M-6VXX complex with quercetin, luteolin, and wogonin based on this binding model were −34.5920 kcal mol^−1^, −32.8415 kcal mol^−1^, and −30.5083 kcal mol^−1^, respectively. The analysis results of the binding model showed that although quercetin had the highest binding energy, it did not directly interact with the key amino acids that formed bonds between IL6R/IL6/IL6ST and Spike. In contrast, Van der Waals interaction between wogonin and the key amino acids Trp291 (IL6ST) and Asp198 (Spike) which formed stable bonds between IL6R/IL6/IL6ST and Spike. (See Figure 5B–5D)

## DISCUSSION

Based on previous studies, the present study improved the analysis method of network pharmacology and molecular docking, and selected the core target in the network based on the maximum degree value, which indicated the role of nodes in network pharmacology. The clusterProfiler package included in the R language was used to obtain the latest Gene Ontology and KEGG pathway for the core target to predict the mechanism of the inhibitory effect of LHQW on the COVID-19 inflammatory stress response. The core target and its receptor complex obtained by network pharmacology were first attached to the S protein by protein-protein docking, and then to the active ingredients connected with the core target by protein-small molecule docking, in order to simulate the process of drug intervention after COVID-19 invades the host cell receptor. These analyses validate the results of network pharmacology and provide a molecular basis for investigating the mechanism of the inhibition of the COVID-19 inflammatory stress response by LHQW.

COVID-19 infection causes a type of lung collateral disease that can be treated using TCM. Its clinical and pathological features include immune system disorders, damage to the deep airway and alveoli caused by an inflammatory reaction, and severe pulmonary congestion. According to the literature [12–14], patients with COVID-19 show symptoms of an excessive inflammatory response, especially in severe cases. Excess release of cytokines and chemokines (IL6, TNF-α, IL2, IL7, CCL2, CXCL10, MCP-1) cause cytokine storm (CS) in these patients. As a result, there is a diffuse damage caused to the lung endothelium and alveolar epithelial cells, leading to acute respiratory distress syndrome (ARDS) and multiple organ dysfunction syndrome (MODS). In the advanced stage of viral infection, CS is considered one of the most important factors of the death in COVID-19 patients.

CS, also known as Cytokine Release Syndrome, is an overactive immune response caused by an external stimuli, as a result of the rapid release of cytokines such as Interleukin, Interferon (IFN), TNF, and Colony-Stimulating Factor (CSF) in large quantities [15–17]. By analyzing 99 confirmed cases of COVID-19, Chen et al. proposed that the virus could infect other cells through the respiratory mucosa and induce a CS, causing severe immune injury to the lungs and other organs [17]. In our study, we found that the core pathogenesis target of COVID-19 was TNF, while the secondary pathogenesis targets included IL6, IL10, and IL2 cytokines. The analysis results were consistent with the above clinical findings and indicated that inflammatory cytokines played crucial roles in COVID-19 infections. Therefore, inhibition of CS is critical for the treatment of COVID-19.

Moreover, we identified the core target IL6 and other secondary targets, such as MAPK8, CXCL8, TP53, MAPK3, IL10, and CASP3, by mapping 45 junction targets, although the core therapeutic target of LHQW AKT1 did not map with the core target TNF of COVID-19. This conclusion is consistent with recent clinical findings. Recent clinical studies have shown a significant increase in the levels of IL6, TNF, and many other inflammatory factors, especially in patients with severe pneumonia. Disease severity was positively correlated with IL6 levels. Once the disease is under control, IL6 levels also decrease rapidly. Notably, if IL6 levels do not decrease rapidly after treatment, the patient prognosis is often poor [12, 18, 19]. Therefore, early detection of IL6 may provide an early indication of severe infection. In addition, IL6 has been identified as a clinical early warning indicator for severe and critically ill patients in the diagnosis and treatment protocol for COVID-19 (Trial Version 8) [6]. Therefore, it is suggested that LHQW may regulate CS by inhibiting IL6, controlling the immunological stress caused by COVID-19, and relieving inflammatory symptoms caused by pulmonary infection.

The biological process involving response to LPS was highlighted in GO enrichment analysis. LPS can cause damage to alveolar epithelial cells and capillary endothelial cells and then change the intercellular space and permeability, causing inflammatory cells to release IL6. It has been reported that IL6 levels in the LPS group were significantly higher than those in the control group (*P* < 0.01), and LHQW could reduce IL6 levels in mouse lung tissue infected by the influenza virus, suggesting that LHQW may alleviate lung injury by inhibiting LPS and decreasing the level of IL6 [20].

The AGE-RAGE signaling pathway based on IL6 was significantly involved in the mechanisms of LHQW treatment of COVID-19 in KEGG pathway analysis. The AGE-RAGE signaling pathway is often activated in diabetic complications, which mediate the downstream activation of a large number of inflammatory cytokines. It has been reported that advanced glycation end product (AGE)/receptor AGE (RAGE)/nuclear transcription factor-κB (NF-κB) signaling pathway played an important role in oxidative stress and excessive inflammatory responses. RAGE is expressed at a low level under normal or physiological conditions, but AGE levels increase during inflammation or trauma and further lead to the upregulation of RAGE expression. Overexpression of RAGE induces the expression and release of a large number of proinflammatory cytokines, such as adhesion molecules, growth factors, IL6, IL8, and TNF-α, subsequently causing tissue injury [21]. A recent study showed that diabetic complications and COVID-19 infection could both activate the AGE-RAGE signaling pathway and promote the progression of COVID-19 [18]. Therefore, LHQW may have a good therapeutic effect on COVID-19 patients with a history of diabetes mellitus.

It has been reported that the invasion of COVID-19 into human cells may be due to ACE2 binding to the receptor-binding domain (RBD) region of the S protein, resulting in membrane fusion and entry of the virus [22, 23]. Single-cell sequencing data, however, indicated that the overall ACE2 expression level was low in various human tissues, especially in pulmonary and bronchial tissues [24, 25]. In addition, recent studies have revealed several neutralizing antibodies, such as mAbs (4A8, COVA1-03, COVA1-21) that bound to the S protein, but the binding site was not in RBD [26, 27]. These results indicate the possibility of other receptors or co-receptors binding to the different domains of the S protein to promote the COVID-19 entry into the cell. According to our protein-protein docking results, IL6R/IL6/IL6ST and Spike were bound stably via conventional hydrogen bonds between the N-terminal domain (NTD) region of the Spike protein S1 subunit and IL6ST. NTD in the S1 subunit plays an important role in recognizing and binding receptors on the surface of the target cell membrane [10, 11]. Therefore, IL6ST is involved in the process by which COVID-19 enters the cell.

Interleukin 6 (IL6, the core target) is a multifunctional cytokine that can carry out signal transduction and plays its biological function only by forming the IL6/IL6R/IL6ST hexameric complex with its receptor. However, under physiological conditions, IL6ST cannot bind to IL6 directly. Only after IL6R binds to IL6 it can bind to IL6ST with high affinity to activate downstream signaling. Thus, IL6 expression can be regulated at the receptor level. Any measure and methods that can affect the binding of IL6 and IL6R or the signal transduction mediated by the binding of IL6/IL6R and IL6ST may be applied in the treatment of IL6-related diseases. Targeting the IL6 receptor to develop and screen drugs is a potential approach for treating IL6-related diseases. Although some artificially synthesized antagonists of IL6 and IL6R have been developed, there are only a few reports on the screening of antagonists from natural products [28]. Conversely, IL6ST, also known as membrane glycoprotein 130, is a shared signal-transducing receptor for a family of more than 10 different four-helix-bundle cytokines, including IL6, leukemia inhibitory factor, ciliary neurotrophic factor (CNTF), oncostatin-M (OSM), and others [29–31].

Therefore, because of the biological characteristics of IL6R/IL6/IL6ST and Spike, this study designed a protein-small molecule docking analysis based on two different binding models (IL6R/IL6 and IL6ST; IL6R/IL6/IL6ST and Spike). The results showed that all three active ingredients (luteolin, quercetin, and wogonin) had perfect CDOCKER interaction energy with the 1P9M-6VXX complex (1P9M: IL6R/IL6/ IL6ST; 6VXX: Spike) based on these two different binding models, with the highest CDOCKER interaction energy found for quercetin. Meanwhile, the analysis results of the IL6R/IL6 and IL6ST binding models indicate that quercetin may affect IL6 signal transduction mediated by the binding of IL6R/IL6 and IL6ST to Gln124 (IL6R/IL6) and His145 (IL6ST). Nevertheless, wogonin may directly influence IL6ST-mediated cytokine signal transduction by competitively binding to Trp291 (IL6ST) and Asp198 (Spike) according to the binding model of IL6R/IL6/IL6ST and Spike.

In this study, we screened for potential targets using network pharmacology, constructing PPI network, and exploring potential pathways by an enrichment analysis, to study the mechanism of protective action of LHQW against COVID-19 symptoms. Furthermore, verification based on molecular docking, which was developed to understand the mechanism and provide explanation for the effectiveness of LHQW for COVID-19 treatment, confirmed the importance of LHQW in the prevention and control of COVID-19 infection. Moreover, because of the use of computer simulation in this study, the potential risk of biosafety was avoided, and the efficiency of the research on sudden infectious diseases and TCM modernization was improved, thereby providing more scientific evidence for the clinical application of LHQW and direction for in-depth research. In addition, this study provides insight into prescription selection for the clinical treatment of COVID-19 and a reference for reducing the risk of the associated clinical CS. Our study is also expected to provide lead compounds for the development of new drugs for the treatment of COVID-19.

In conclusion, this study revealed that the NTD region of the COVID-19 Spike protein had a high CDOCKER interaction energy with IL6ST using network pharmacology and molecular docking analysis. Quercetin in LHQW may directly affect the interaction between IL6R/IL6 and IL6ST by competitively binding to Gln124 (IL6R/IL6) and His145 (IL6ST) to mediate IL6R/IL6/IL6ST signal transduction. Wogonin may directly influence the interaction between IL6R/IL6/IL6ST and Spike via competitive binding to Trp291 (IL6ST) and Asp198 (Spike), affect IL6ST-mediated cytokine signal transduction, and regulate the excessive inflammatory stress caused by CS. LHQW may inhibit the LPS-mediated inflammatory response and regulate the AGE-RAGE signaling pathway by regulating the function of IL6, which in turn can influence oxidative stress, excessive inflammatory response, and immune function to control the inflammatory stress response during COVID-19 infection. Due to the limitations of the computational methods of chemistry and biology, the results of this study need to be verified by follow-up experiments to provide a basis for the treatment of COVID-19 using TCM.

## METHODS

### Database and software

The following databases and software packages were used in this study: GeneCards [32] (https://www.genecards.org/), Traditional Chinese Medicine Systems Pharmacology Database [33] (TCMSP, http://tcmspw.com/tcmsp.php), UniProt [34] (https://www.uniprot.org/), STRING [35] (https://STRING-db.org/cgi/input.pl), PubChem [36] (https://pubchem.ncbi.nlm.nih.gov/), RCSB Protein Data Bank [37] (http://www.rcsb.org/), R(version R3.6.1) [38], Cytoscape (3.7.2) [39], ZDOCK Server 3.0.2 [40], Discovery Studio 2019 Client, Pymol (version 2.4.1).

### Network pharmacology and molecular docking analysis of the mechanism of LHQW in treating COVID-19

The detailed information is available in the Flow Charts 1 and 2.

**Flow Chart 1:**
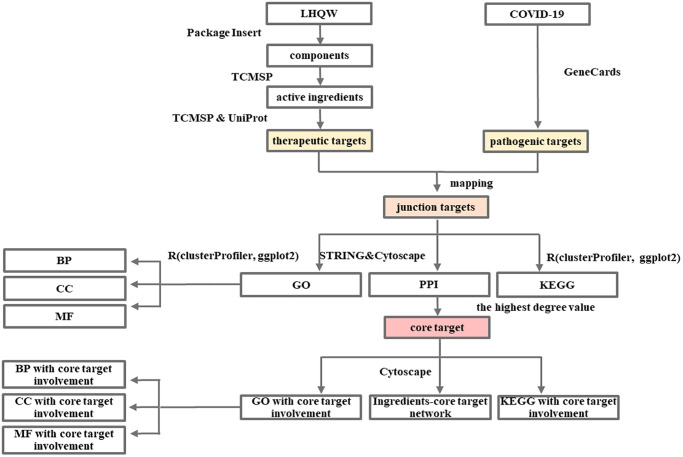
Network pharmacology-based analysis of the mechanism of LHQW in treating COVID-19.

**Flow Chart 2:**
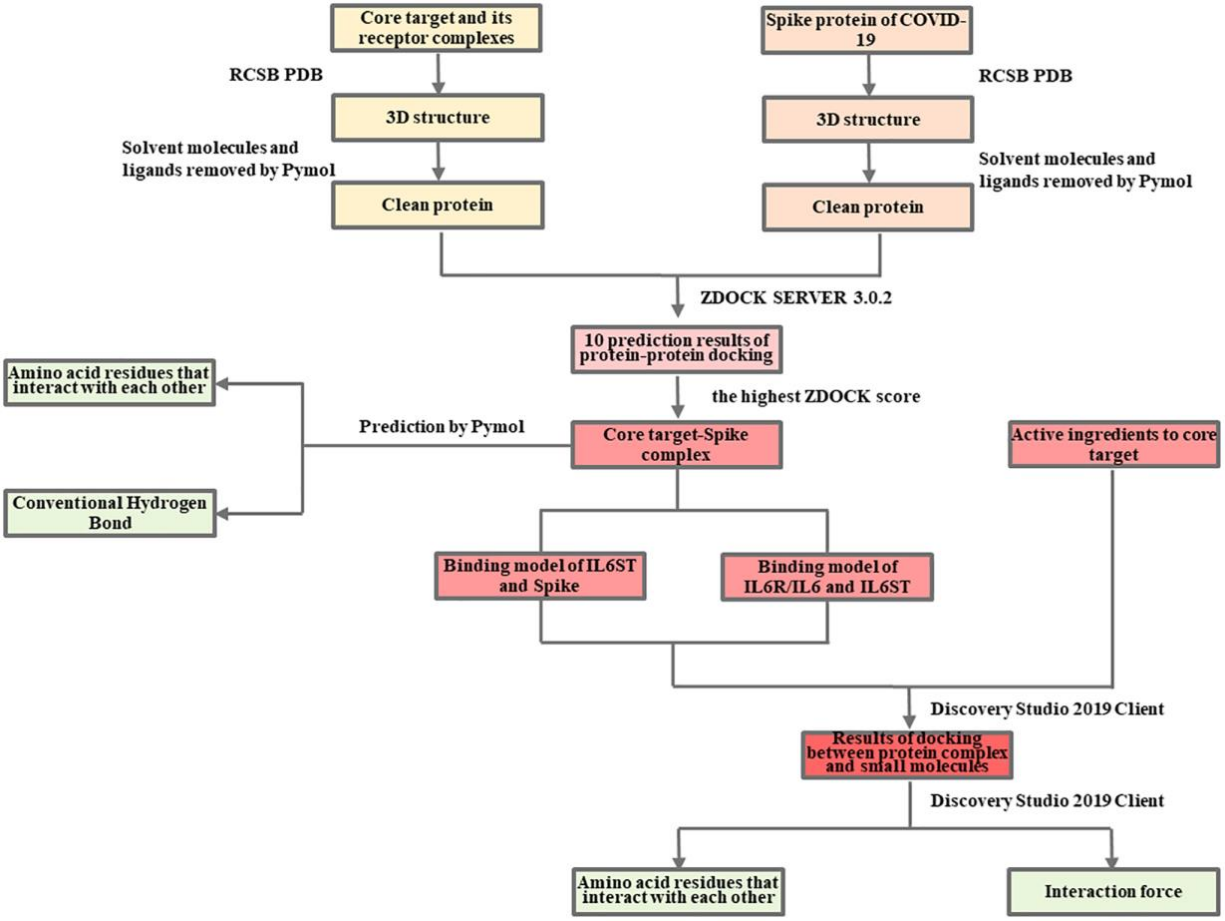
Molecular docking analysis of the mechanism of LHQW in treating COVID-19.

### Network pharmacology of the mechanism of LHQW in treating COVID-19

#### Screening of COVID-19 pathogenic targets, LHQW active ingredients and therapeutic targets

A search was conducted on the GeneCards database using the keywords “coronavirus disease 2019,” “coronavirus pneumonia,” “coronavirus” and “novel coronavirus 2019.” The results were exported to Microsoft Excel. For the purposes of this study, those with a score ≥1 were defined as pathogenic targets of COVID-19. Information has been added according to the literature.

From LHQW components (Supplementary Table 1), in the TCMSP, the main active ingredients were screened according to oral bioavailability (OB) ≥30% and drug-likeness (DL) ≥0.18, and the therapeutic targets of the main active ingredients were screened through Targets information of TCMSP. Finally, gene symbol conversion was performed on the therapeutic targets screened in TCMSP according to UniProt. Additional information has been added to the literature.

#### Protein-Protein Interaction (PPI) network of COVID-19 pathogenic targets, LHQW therapeutic targets, junction targets of COVID-19 and LHQW and LHQW ingredients-targets network

COVID-19 pathogenic targets, LHQW therapeutic targets, and junction targets of COVID-19 and LHQW through mapping were imported into the STRING database and optimized using Cytoscape, respectively. The degree value was calculated using a network analyzer. The degree value is an indicator of the importance of network nodes. The higher the value is, the stronger is the correlation between the corresponding node and other nodes. Subsequently, PPI-related information was visualized through a network diagram, and the core target of COVID-19 pathogenic targets, LHQW therapeutic targets, and junction targets were extracted based on the maximum value of degree, separately.

Cytoscape was used to introduce the active ingredients and potential therapeutic targets of LHQW, and the ingredients-targets network was constructed.

#### Gene-Concept network of gene ontology enrichment analysis and KEGG pathway analysis of the junction targets of COVID-19 and LHQW

GO includes three following aspects: biological process, cellular component, and molecular function. GO enrichment and Kyoto Encyclopedia of Genes and Genomes (KEGG) pathway analyses were conducted for junction targets of COVID-19 and LHQW using the R and clusterProfiler [41] package in this study, respectively. In addition, the ggplot2 [42] package was used to plot the Gene-Concept network to identify the biological process, the location of the reaction in the cell, the molecular function involved, and relevant KEGG pathway of LHQW in treating COVID-19, separately.

#### Network of active ingredients, GO and KEGG pathways corresponding to the core target

The core target, corresponding GO and KEGG pathways, and active ingredients were imported into Cytoscape, respectively, to build the network.

### Verification of molecular docking for LHQW in treating COVID-19 by the results of network pharmacology

According to the results of network pharmacology, IL6 is the core target of LHQW in treating COVID-19, and the active ingredients of LHQW, luteolin, quercetin, and wogonin are all connected to the core target IL6. Therefore, this study will carry out further molecular docking verification based on these results.

#### Protein-protein docking analysis between IL6R/IL6/IL6ST complex and COVID-19 spike

RCSB PDB databases were used to download the 3D structure PDB files of IL6R/IL6/IL6ST complex (PDB ID: 1P9M) and Spike protein (PDB ID: 6VXX), respectively, and then imported into Pymol to remove solvent molecules and ligands. After that, they were imported into ZDOCK SERVER 3.0.2 (Fast Fourier transform-based protein docking program) for protein-protein docking, and all possible binding models in the translation and rotation space between the two proteins were searched. The ZDOCK score was calculated using root-mean-square deviation (RMSD). Based on the maximum ZDOCK score, the optimal attitude of the two bindings was screened, and the output was the 1P9M-6VXX (IL6R/IL6/IL6ST-Spike) complex. The amino acid residues and hydrogen bonding forces of protein-protein binding were predicted using Pymol.

#### Protein-small molecule docking analysis

CDOKER [43] is a high-accuracy molecular docking method based on the CHARMm force field. In this method, high-temperature kinetics are used to search for the flexible conformation of the ligand molecules. It is believed that the lower the CDOCKER interaction energy, the more stable the conformation of the ligand binding to the receptor. In addition, ≤−5.0 kcal mol^−1^ indicates that the protein and small molecule can bind, and ≤−7.0 kcal mol^−1^ indicates strong binding ability. The 1P9M-6VXX complex was introduced into the Discovery Studio 2019 Client, and polar hydrogens were added to the complex via Add Polar. According to the results of the network pharmacology and the characteristics of IL6 and Spike in physiological conditions, the active sites between IL6R/IL6 and IL6ST, between IL6R/IL6/IL6ST and Spike protein were identified from Receptor Cavities, and the Apply Forcefield was used to force field to the complex. The PubChem database was used to obtain the SDF files of the active ingredients to the core target IL6 as small molecules, which were imported into the Discovery Studio 2019 Client. The Apply Forcefield was used to generate the force field to the small molecules. Finally, the Dock Ligands (CDOCKER) was used for protein-small molecule docking, adjusting the Top Hits-Pose Cluster Radius to 0.5, and the other parameters were kept at default.

### Availability of data and materials

Publicly available datasets were analyzed in this study. These data can be found here: [GeneCards] at (https://www.genecards.org/), [Traditional Chinese Medicine Systems Pharmacology Database (TCMSP)] at (http://tcmspw.com/tcmsp.php), [UniProt] at (https://www.uniprot.org/), [STRING] at (https://STRING-db.org/cgi/input.pl), [PubChem] at (https://pubchem.ncbi.nlm.nih.gov/), [RCSB Protein Data Bank] at (http://www.rcsb.org/).

## Supplementary Materials

Supplementary Figures

Supplementary Tables
