# Supplementary material for: New tale on LianHuaQingWen: IL6R/IL6/IL6ST complex is a potential target for COVID-19 treatment
Source: Aging (Albany NY). 2021 Nov 3;13(21):23913–35. doi: 10.18632/aging.203666 (PMC8610116; doi:10.18632/aging.203666)
Supplement: Supplementary Figures [file aging-13-203666-s001.pdf]

SUPPLEMENTARY FIGURES

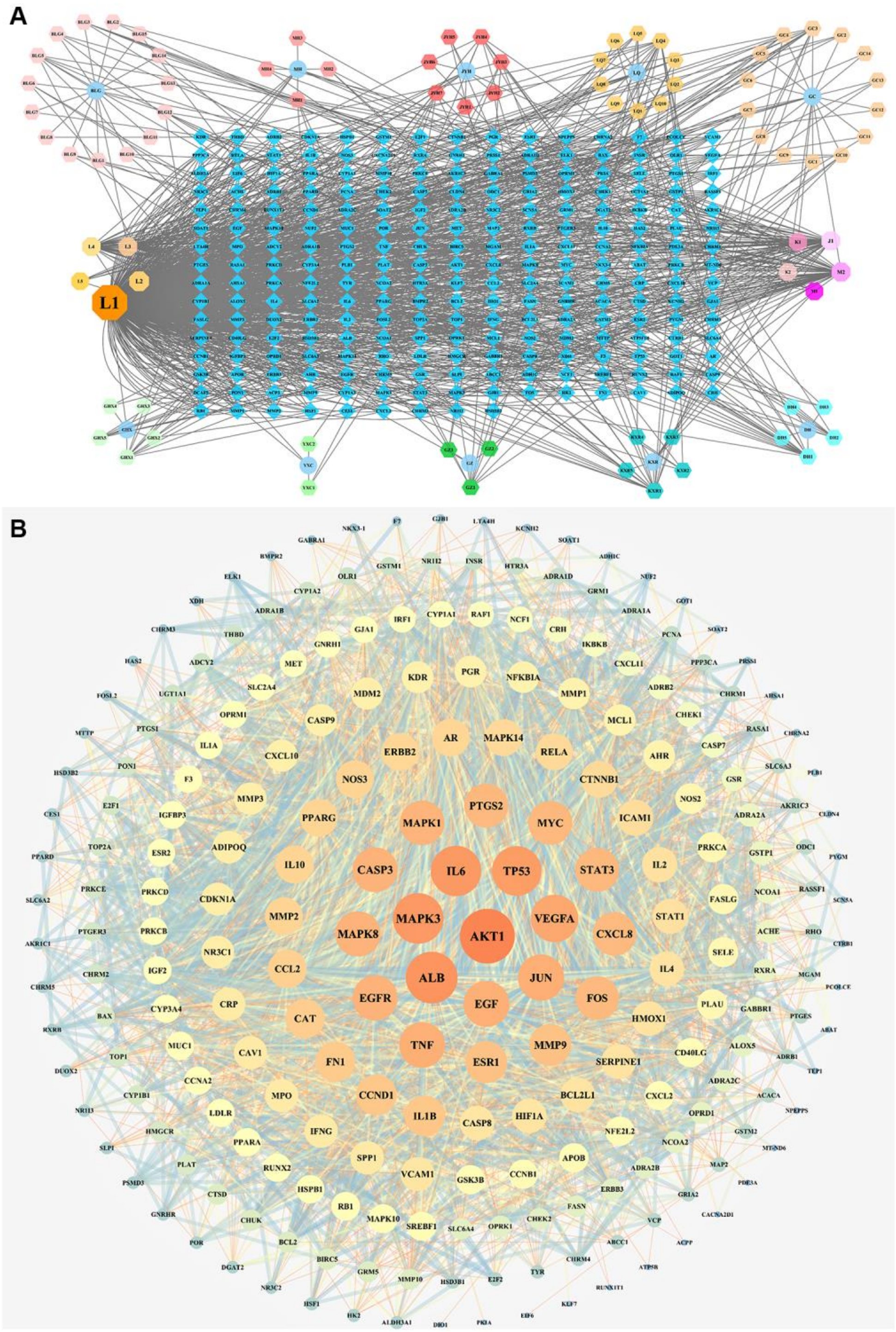

Supplementary Figure 1. Network of LHQW active ingredients and potential targets and PPI network of targets. (**A**) Network of LHQW active ingredients and targets. (**B**) PPI network for potential therapeutic targets of LHQW.

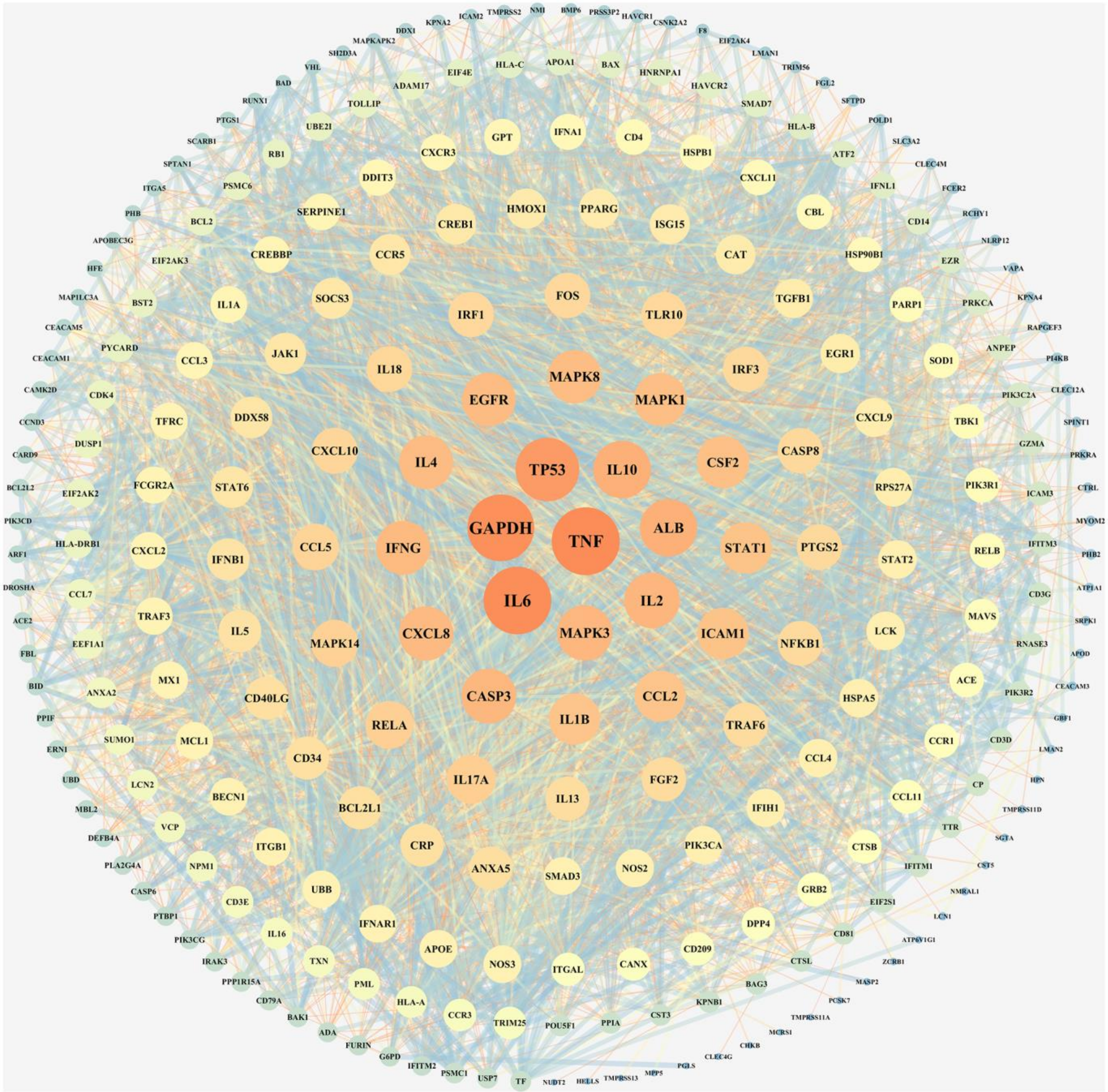

Supplementary Figure 2. PPI network for potential pathogenic targets of COVID-19.

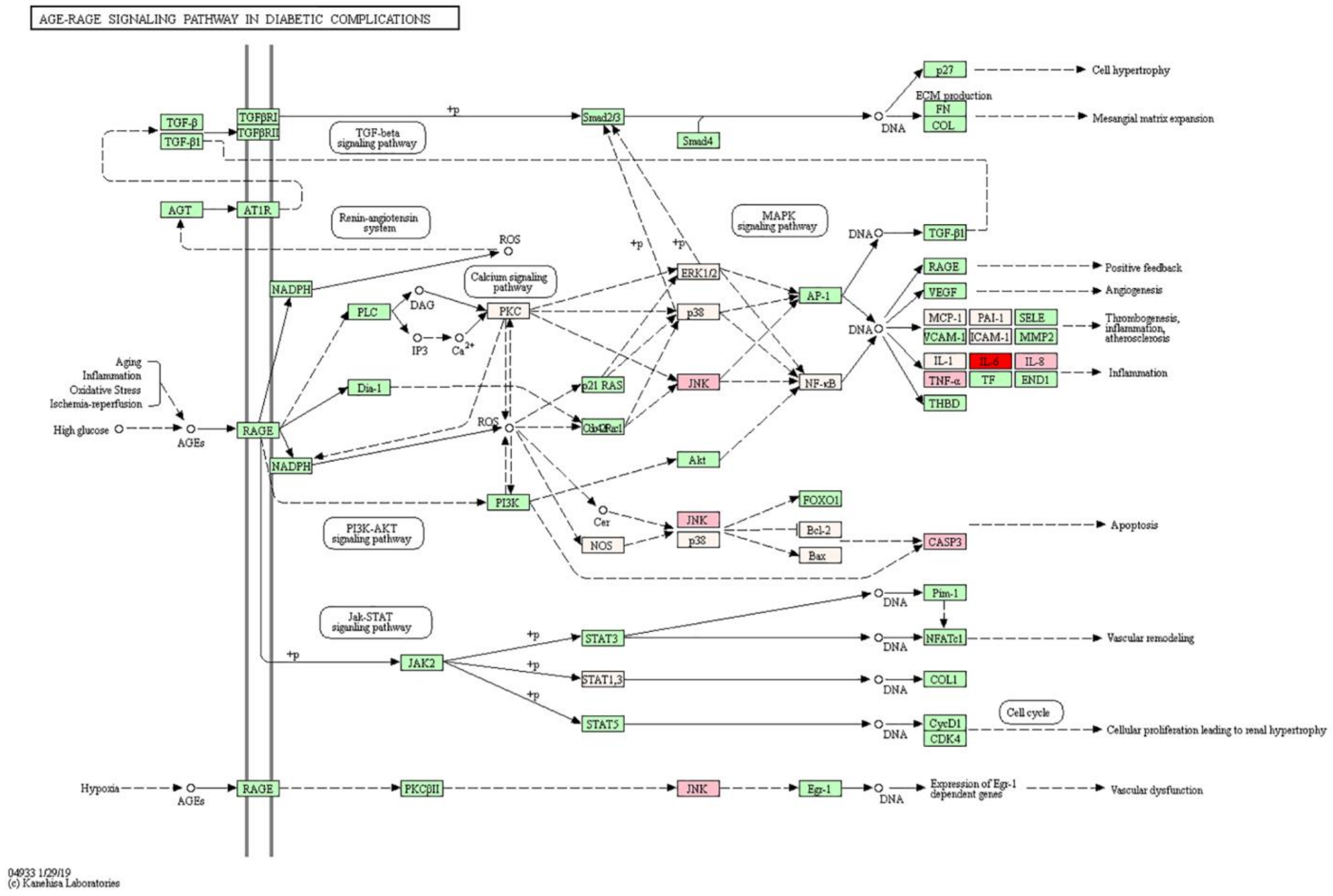

Supplementary Figure 3. AGE-RAGE signaling pathway in diabetic complications. The pathway contains 19 targets. Including the core target IL6 (red), five secondary targets (pink), six tertiary targets (seashell), and seven quaternary targets (seashell).
