# Supplementary material for: New tale on LianHuaQingWen: IL6R/IL6/IL6ST complex is a potential target for COVID-19 treatment
Source: Aging (Albany NY). 2021 Nov 3;13(21):23913–35. doi: 10.18632/aging.203666 (PMC8610116; doi:10.18632/aging.203666)
Supplement: Supplementary Tables [file aging-13-203666-s002.pdf]

SUPPLEMENTARY TABLES

Supplementary Table 1. LHQW Components.

Components	Amount	Category	Efficacy
Forsythiae Fructus	255 g	Primary components	They expel "wind" and clear away heat clear away heat and toxic materials.
Lonicerae Japonicae Flos	255 g		
Ephedra Herba	85 g	Secondary components	Ephedra Herba frees lung and relieves asthma; Gypsophyte expels the wind; Amygdalus Communis Vas relieves cough and asthma.
Gypsum	255 g		
Amygdalus Communis Vas	85 g		
Isatidis Radix	255 g	Adjuvants	They clear away the "lung heat", resolve dampness of the lung, resolve dampness of the lung, regulate the flow of vital energy and remove obstruction to it, strengthen the spleen and stomach.
Fortunes Boss Fern Rhizome	255 g		
Houttuyniae Herba	255 g		
Menthol crystal	7.5 g		
Pogostemon Cablin (Blanco) Benth.	85 g		
Radix Rhei Et Rhizome	51 g		
Rhodiola rosea L.	85 g		
licorice	85 g		

Supplementary Table 2. Components- Active Ingredients- ID.

Components	Active Ingredients	ID
Forsythiae Fructus<br>Lonicerae Japonicae Flos<br>Ephedra Herba<br>Houttuyniae Herba<br>Pogostemon Cablin (Blanco) Benth.<br>licorice	quercetin	L1
Forsythiae Fructus<br>Lonicerae Japonicae Flos<br>Ephedra Herba<br>Fortunes Boss Fern Rhizome<br>Houttuyniae Herba<br>licorice	kaempferol	L2
Forsythiae Fructus<br>Lonicerae Japonicae Flos<br>Ephedra Herba<br>Isatidis Radix<br>Radix Rhei Et Rhizome	beta-sitosterol	L3
Forsythiae Fructus<br>Lonicerae Japonicae Flos<br>Ephedra Herba	luteolin	L4
Forsythiae Fructus<br>Amygdalus Communis Vas<br>licorice	Mairin	L5
Lonicerae Japonicae Flos<br>Ephedra Herba<br>Amygdalus Communis Vas<br>Isatidis Radix	Stigmasterol	J1

Ephedra Herba<br>Amygdalus Communis Vas	(+)-catechin	M1
Ephedra Herba<br>licorice	naringenin	M2
Amygdalus Communis Vas<br>licorice	Glycyrol	K1
Amygdalus Communis Vas<br>licorice	Licochalcone B	K2
Forsythiae Fructus	(−)-Phillygenin	LQ1
Forsythiae Fructus	ACon1_001697	LQ2
Forsythiae Fructus	Onjixanthone I	LQ3
Forsythiae Fructus	bicuculline	LQ4
Forsythiae Fructus	(2R,3R,4S)-4-(4-hydroxy-3-methoxy-phenyl)-7-methoxy-2,3-dimethylol-tetralin-6-ol	LQ5
Forsythiae Fructus	(+)-pinoresinol monomethyl ether	LQ6
Forsythiae Fructus	(3R,4R)-3,4-bis[(3,4-dimethoxyphenyl)methyl]oxolan-2-one	LQ7
Forsythiae Fructus	arctiin	LQ8
Forsythiae Fructus	3beta-Acetyl-20,25-epoxydammarane-24alpha-ol	LQ9
Forsythiae Fructus	wogonin	LQ10
Lonicerae Japonicae Flos	(−)-(3R,8S,9R,9aS,10aS)-9-ethenyl-8-(beta-D-glucopyranosyloxy)-2,3,9,9a,10,10a-hexahydro-5-oxo-5H,8H-pyrano[4,3-d]oxazolo[3,2-a]pyridine-3-carboxylic acid_qt	JYH1
Lonicerae Japonicae Flos	Ioniceracetalides B_qt	JYH2
Lonicerae Japonicae Flos	Centauroside_qt	JYH3
Lonicerae Japonicae Flos	5-hydroxy-7-methoxy-2-(3,4,5-trimethoxyphenyl)chromone	JYH4
Lonicerae Japonicae Flos	ZINC03978781	JYH5
Lonicerae Japonicae Flos	Eriodyctiol (flavanone)	JYH6
Lonicerae Japonicae Flos	beta-carotene	JYH7
Ephedra Herba	eriodictyol	MH1
Ephedra Herba	taxifolin	MH2
Ephedra Herba	Truflex OBP	MH3
Ephedra Herba	Genkwanin	MH4
Amygdalus Communis Vas	l-SPD	KXR1
Amygdalus Communis Vas	Machiline	KXR2
Amygdalus Communis Vas	Phaseol	KXR3
Amygdalus Communis Vas	liquiritin	KXR4
Amygdalus Communis Vas	estrone	KXR5
Isatidis Radix	3-[[(2R,3R,5R,6S)-3,5-dihydroxy-6-(1H-indol-3-yloxy)-4-oxooxan-2-yl]methoxy]-3-oxopropanoic acid	BLG1
Isatidis Radix	neohesperidin_qt	BLG2
Isatidis Radix	(*E*)-3-(3,5-dimethoxy-4-hydroxyb-enzylidene)-2-indolinone	BLG3
Isatidis Radix	2-(9-((3-methyl-2-oxopent-3-en-1-yl)oxy)-2-oxo-1,2,8,9-tetrahydrofuro[2,3-h]quinolin-8-yl)propan-2-yl acetate	BLG4
Isatidis Radix	hydroxyindirubin	BLG5
Isatidis Radix	Isaindigodione	BLG6
Isatidis Radix	(*E*)-2-[(3-indole)cyanomethylene-]-3-indolinone	BLG7
Isatidis Radix	3-[(3,5-dimethoxy-4-oxo-1-cyclohexa-2,5-dienylidene)methyl]-2,4-dihydro-1H-pyrrolo[2,1-b]quinazolin-9-one	BLG8
Isatidis Radix	Sinensetin	BLG9
Isatidis Radix	Sinoacutine	BLG10
Isatidis Radix	6-(3-oxoindolin-2-ylidene)indolo[2,1-b]quinazolin-12-one	BLG11
Isatidis Radix	acacetin	BLG12
Isatidis Radix	quindoline	BLG13
Isatidis Radix	isovitexin	BLG14
Isatidis Radix	Dinatin	BLG15

Fortunes Boss Fern Rhizome	ZINC00035529	GZ1
Fortunes Boss Fern Rhizome	11-Hydroxynumantenine	GZ2
Fortunes Boss Fern Rhizome	(2R)-5,7-dihydroxy-2-(4-hydroxyphenyl)chroman-4-one	GZ3
Houttuyniae Herba	Spinasterol	YXC1
Houttuyniae Herba	Isoramanone	YXC2
Pogostemon Cablin (Blanco) Benth.	5-Hydroxy-7,4′-dimethoxyflavanon	GHX1
Pogostemon Cablin (Blanco) Benth.	quercetin 7-*O*-β-D-glucoside	GHX2
Pogostemon Cablin (Blanco) Benth.	Diop	GHX3
Pogostemon Cablin (Blanco) Benth.	phenanthrone	GHX4
Pogostemon Cablin (Blanco) Benth.	irisolidone	GHX5
Radix Rhei Et Rhizome	aloe-emodin	DH1
Radix Rhei Et Rhizome	EUPATIN	DH2
Radix Rhei Et Rhizome	(−)-catechin	DH3
Radix Rhei Et Rhizome	rhein	DH4
Radix Rhei Et Rhizome	Toralactone	DH5
licorice	7,2′,4′-trihydroxy-5-methoxy-3-arylcoumarin	GC1
licorice	licopyranocoumarin	GC2
licorice	shinpterocarpin	GC3
licorice	Vestitol	GC4
licorice	Glyasperins M	GC5
licorice	(2R)-7-hydroxy-2-(4-hydroxyphenyl)chroman-4-one	GC6
licorice	formononetin	GC7
licorice	Glypallichalcone	GC8
licorice	Licoagrocarpin	GC9
licorice	isorhamnetin	GC10
licorice	Medicarpin	GC11
licorice	7-Methoxy-2-methyl isoflavone	GC12
licorice	licochalcone a	GC13
licorice	sitosterol	GC14
